# Regulation of HSC development and function by Lin28b

**DOI:** 10.3389/fcell.2025.1555877

**Published:** 2025-03-12

**Authors:** Grant Cox, Michihiro Kobayashi, Brian D. Rudd, Momoko Yoshimoto

**Affiliations:** ^1^ Department of Neurology, University of Washington, Seattle, WA, United States; ^2^ Department of Investigative Medicine, Western Michigan University Homer Stryker M.D. School of Medicine, Kalamazoo, MI, United States; ^3^ Department of Microbiology and Immunology, Cornell University, Ithaca, NY, United States

**Keywords:** fetal and adult hematopoietic stem cells, HSC expansion, LIN28B, Let7, HMGA2, IGF2BP2

## Abstract

Hematopoietic stem cells (HSCs) provide all kinds of blood cells for life while maintaining self-renewal ability. During development, HSCs are first produced in the mouse embryo around embryonic day (E) 11. At this time, only one or two transplantable HSCs can be detected per embryo. Then, HSCs migrate to the fetal liver, where the number of HSCs rapidly increases, showing enhanced self-renewal ability. After birth, a transition occurs from the rapidly proliferating fetal HSCs to the more slowly dividing adult HSCs, which ends by 3–4 weeks of age. It is known that fetal HSCs express distinct surface markers and transcriptomes and produce a variety of distinct immune cells that are not made by adult HSCs. Accumulating evidence indicates that the ontogeny of the hematopoietic system is driven by a highly conserved and developmentally regulated RNA binding protein known as *Lin28b*. *Lin28b* is predominantly expressed in the fetal hematopoietic stem and progenitor cells (HSPCs) and regulates the developmental switch from fetal to adult HSCs. In this review, we will provide an overview of how *Lin28b* regulates the expansion and differentiation of HSCs in early life. These insights can be taken into consideration when developing *ex vivo* HSC expansion utilizing such physiological characteristics of HSCs.

## Introduction

*Ex vivo* HSC expansion is a longstanding desire in the hematology field, since it would enable more efficient transplantation therapy with larger numbers of HSCs. However, it is challenging to expand HSCs. First, HSCs seldomly divide. In fact, the fetal to neonatal stage is the only time when HSCs expand in number. Second, once HSCs are transferred to an *in vitro* environment, they either quickly differentiate and lose the ability to self-renew or die without the appropriate signals or environment. Starting from traditional stromal cell cocultures and cytokine combinations, recent advancements using small molecules (such as UM171) and albumin alternatives (such as polyvinyl alcohol) have improved strategies for HSC expansion (Ueda et al., 2000; Fares et al., 2014; Bastani et al., 2023; Rubio-Lara et al., 2023; Sakurai et al., 2023; Meaker and Wilkinson, 2024). However, these methods also induce HSC differentiation, and it is not clear that long-term engrafting HSCs are really expanded (Bastani et al., 2023). The mechanisms of HSC expansion and the potential long-term effects of the cytokines and molecules on HSC function are not well understood. Therefore, it is critically important to better understand the normal physiological settings in which HSCs expand. Notably, self-renewing HSCs expand considerably in the fetal liver (Ema and Nakauchi, 2000), while their differentiation is minimal (Yokomizo et al., 2022; Kobayashi et al., 2023). Thus, understanding the mechanism of HSC expansion in the fetus is key for translating *ex-vivo* HSC expansion. In this paper, we will provide an overview of the mechanisms by which HSCs expand in early life and explain the biological differences between fetal and adult HSCs. Also, we will address how manipulation of HSCs into “fetal-type HSCs” impacts on hematopoiesis and their differentiation capability, specifically focusing on *Lin28b* expression, which regulates the function of fetal HSCs. A better understanding of these mechanisms will provide us with key information needed to improve HSC expansion strategies.

## Development of HSCs in the mouse embryo

HSCs have the ability to self-renew and differentiate into multiple lineages, allowing them to produce all types of blood cells and maintain blood homeostasis for life. While HSCs are first produced from special endothelial cells (ECs), referred to as hemogenic ECs, in the aortic region of the embryo around E11.5, multiple waves of hematopoiesis proceed before HSC emergence (Medvinsky and Dzierzak, 1996; Chen et al., 2009; Kobayashi and Yoshimoto, 2023). As early as E7.5, primitive type erythroid and myeloid cell production occurs in the extraembryonic yolk sac (YS), followed by definitive type erythro-myeloid progenitor (EMP) production (Lux et al., 2008). At E8.5, lymphoid and multipotent progenitor potentials can be detected in both the YS and the para-aortic region (Godin et al., 1995; Cumano et al., 1996; Nishikawa et al., 1998; Yoshimoto et al., 2011; Yoshimoto et al., 2012). The precursors of HSCs (pre-HSCs) that can become transplantable after aggregation culture are detectable around E10.5–11.5 (Rybtsov et al., 2011; Kobayashi et al., 2019). At this time, the pool of pre-HSCs is comprised of various hematopoietic progenitors that arise from hemogenic ECs, including HSC-independent multipotent progenitors (Dignum et al., 2021; Kobayashi et al., 2023). Some of the pre-HSCs mature into HSCs in the fetal liver, where they proliferate and then migrate to the spleen and bone marrow just before birth. After birth, HSCs are maintained in the bone marrow to provide blood homeostasis for life.

HSCs are defined by transplantation assays, specifically as cells that repopulate the hematopoietic system in irradiated mice. Using transplantation assays, several groups have examined the numbers of HSCs found throughout gestation. Kumaravelu et al. reported that there was only one HSC in the YS, aorta-gonad-mesonephros (AGM) region, and fetal liver at E11.5 (Kumaravelu et al., 2002). At E12.5, they detected approximately two to 3 HSCs in the YS, AGM region, and circulation. However, interestingly, 53 HSCs were detected in the fetal liver at E12.5, indicating a rapid increase in total HSC numbers per embryo. At E13.5, the numbers of HSCs in the YS and AGM region diminished, but the number of HSCs in the fetal liver increased to 260, which is an astonishing 5-fold increase from E12.5. Additionally, the placenta has been reported to contain significant numbers of HSCs at E12.5 – E13.5 (Gekas et al., 2005). Ema et al. reported that HSCs further proliferate in the fetal liver during E12.5 to E16.5 (Ema and Nakauchi, 2000). Based on all these findings, we can estimate that HSCs undergo 1.8 divisions from E12.5 to 14.5, and 3.3 divisions from E12.5 to 16.5.

## Cell cycle and engraftment capacity

As mentioned above, while most adult HSCs in the bone marrow are quiescent, which divide only once every 2–5 months, HSCs in the fetus are in cell cycle, which allows them to expand during the transition from the aortic region to the fetal liver, and from the fetal liver to the bone marrow (Cheshier et al., 1999; Wilson et al., 2008). When pre-HSCs arise from ECs at E10.5, half of them are cycling in the G2M phase (Batsivari et al., 2017). Cycling HSCs can be found until ∼3 weeks of age. However, by 4 weeks of age, there is a rapid decrease in proliferation and most of the HSCs are in the resting (G0) phase (Bowie et al., 2006; Copley et al., 2013). This cell cycle change is accompanied by characteristic changes in surface markers (CD34, CD38) (Matsuoka et al., 2001) and gene expression profiles (Sox17, Lin28b) (Kim et al., 2007; Yuan et al., 2012; Copley et al., 2013). How do these cell-cycling statuses and engraftment capabilities correlate?

Batsivari et al. examined the engraftment capabilities of HSCs in different phases of cell-cycling in mid-gestation of the mouse embryo (Batsivari et al., 2017). At E11.5, both cycling and non-cycling (G0/G1) pre-HSCs can engraft in the recipient mice after aggregation culture. In the fetal liver, until E14, the cycling HSCs possess the ability to engraft after transplantation, but after that time, only G0/G1 HSCs exhibit engraftment capability (Batsivari et al., 2017). A report by Bowie et al. precisely examined the repopulating capability of HSPCs based on their cell cycle from E14 fetal liver to up to 10 weeks bone marrow (Bowie et al., 2006). They found a clear transition of HSPC repopulation capability from 3-week bone marrow cells in the G1/S/G2/M phases to 4-week bone marrow cells in the G0 phase. The different results in timing for engraftable cycling HSCs (until E14 vs 3 weeks of age) may be based on which donor population included the G1 phase cells (because G1 phase cells are supposed to engraft efficiently). In any case, what these data suggest is that HSCs enter cell cycle in early life in order to expand but later must go back to G1/G0 status to maintain the ability to engraft.

Another aspect of the developmental changes in engraftment capability may relate to differences in the environment. CXCL12, for example, is a niche factor in the bone marrow that maintains the self-renewal ability of HSCs (Sugiyama et al., 2006). CXCL12 antagonist is usually used to harvest donor peripheral blood stem cells mobilized from the bone marrow (Broxmeyer et al., 2005). Interestingly, Bowie et al. showed that when recipient mice were treated with CXCL12 antagonist, the engraftment capacity of these cycling HSCs from 4 week-old bone marrow was rescued (Bowie et al., 2006), suggesting that it may be advantageous for cycling HSCs not to interact with CXCL12 in the adult bone marrow. However, CXCL12 expression is indispensable for HSC homing to neonatal bone marrow (Ara et al., 2003), and most HSCs are cycling at neonatal stage. It is possible that the structure of neonatal bone marrow is different from the structure of adult bone marrow; thus, the interaction between cycling HSCs and CXCL12 could be different between neonates and adults. Indeed, fetal- and adult-derived HSPCs prefer to engraft in environments of similar ages: fetal HSCs engraft more efficiently in neonatal recipient mice, while adult HSPCs engraft more efficiently in adult recipient mice (Arora et al., 2014).

Consistent with the idea that certain environmental factors may support cycling HSCs, Sigurdsson et al. have shown that bile duct acid can protect cycling HSCs in the fetal liver (Sigurdsson et al., 2016). These studies suggest that the environment plays a pivotal role in protecting cycling HSCs, but further investigation of the role of the environment for HSPCs of different ages is necessary.

## Lin28b regulates the transition from fetal to adult HSCs

Up until now, we have described developmental changes in HSCs that occur during the fetal and neonatal stages of life, including their cell cycle and engraftment capabilities. An important question is how HSC expansion during the fetal and neonatal period is maintained. A number of studies have suggested that the key molecule that distinguishes fetal from adult HSCs is *Lin28b*.

*Lin28* was originally identified in *Caenorhabditis elegans* as a heterochronic gene, which regulates a variety of developmental events, including patterns of cell division, the lengths of specific cell cycles, and stage-specific terminal differentiation events (Ambros and Horvitz, 1984). *Lin28b* encodes an RNA binding protein that binds and inhibits the *Let-7* miRNA family; thus, *Lin28b* expression diminishes *Let-7* expression and absence of *Lin28b* expression increases *Let-7* expression. (Ambros and Horvitz, 1984; Moss et al., 1997; Yuan et al., 2012). *Lin28* plays a critical role during development through the regulation of miRNA biogenesis, transcriptional level DNA regulation, and post-transcriptional direct mRNA binding in various tissues (Polesskaya et al., 2007; Newman et al., 2008; Oshima et al., 2016; Wang et al., 2019). *Lin28-let7* axis is known to regulate glucose metabolism via insluin-PI3K-mTOR pathway (Zhu et al., 2011). *Lin28* regulates proliferation and self-renew of human and mouse embryonic stem cells (Xu et al., 2009). LIN28B can function with NANOG, OCT4, and SOX2 and both LIN28A and LIN28B are required for efficient reprograming of human inducible Pluripotent Stem Cells (iPSCs) (Zhang et al., 2016). As such, Lin28b is expressed in stem cells in various tissues and cancer stem cells (Shyh-Chang and Daley, 2013). In hematopoietic lineages, with several partners, *Lin28b* acts as a heterochronic regulator of the switch from fetal to adult gene expression by their diminished expression within the context of HSC self-renewal, lineage commitment, and differentiation (Yuan et al., 2012).

### Lin28b expression in fetal HSPCs

**FIGURE 1:**
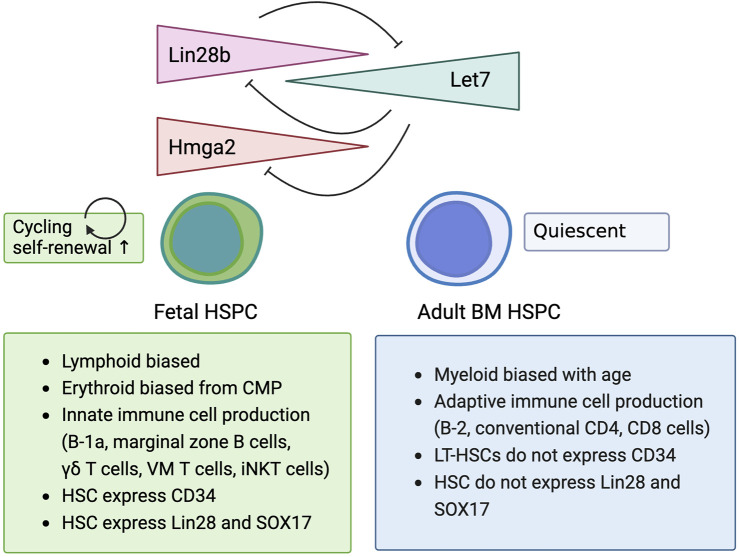
Lin28b regulates biological characteristics of fetal hematopoietic stem/progenitor cell.

*Lin28b* has attracted the attention of the hematology field due to its expression in fetal liver HSCs and its ability to enhance self-renewal (Yuan et al., 2012; Copley et al., 2013). Importantly, when *Lin28b* is overexpressed in adult bone marrow lineage^−^sca-1^+^c-kit^+^ HSPCs (LSK cells) and transplanted into lethally irradiated congenic mice, these *Lin28b*-expressing HSCs differentiate into fetal-type immune cells, including B-1a cells, γδT cells, and NKT cells, which are not usually produced by adult bone marrow HSCs (Yuan et al., 2012; Wang et al., 2019). Yuan et al. showed that overexpression of *Lin28b* reprogrammed adult HSCs into fetal-type HSCs (Figure 1) (Yuan et al., 2012). Wang et al. further examined whether overexpressing *Lin28b in vivo* could generate fetal-like HSCs in adult mice (Wang et al., 2019). For these studies, they used doxycycline inducible *Lin28b* transgenic mice (iLin28b mice) (Zhu et al., 2011). Gene expression profiles of common lymphoid progenitors (CLPs) from the fetal liver, adult bone marrow, and adult iLin28b bone marrow revealed that *Lin28b* overexpression alone might not be sufficient to completely reprogram HSPCs into fetal-type HSCs. The Wang group also identified another RNA binding protein, *Igf2bp3*, that interacts with Lin28b, and these two proteins share multiple target mRNA binding sites. When *Igf2bp3* was retrovirally transduced into iLin28 HSPCs (LSK cells) in the adult bone marrow and transplanted into Rag1^−/−^ mice, better repopulation of fetal-type B-1a and marginal zone B cells resulted, indicating the important role of *Igf2bp3* in HSPC reprogramming (Wang et al., 2019).

### Lin28b and its co-factors enhance the self-renewal ability of HSCs

Among the *Let-7* target genes (*Hmga2, Lin28b, Igf2bp3, Igf2bp2, Igf2bp1*, and *Slc31a1*), *Hmga2* has been found to follow the expression pattern of *Lin28b* over the course of development. The HMGA2 protein is a member of the HMGA family of nonhistone chromatin proteins. HMGA2 proteins have been reported to play critical roles in cell proliferation, cell-cycle progression, apoptosis, senescence, and cancer development (Ikeda et al., 2011). Interestingly, when *Lin28b* levels decrease, *Hmga2* levels in HSCs also decrease, along with their self-renewal ability (Copley et al., 2013). When *Lin28b* is overexpressed in CD45^+^EPCR^+^CD48^−^CD150^+^ (ESLAM) HSCs from the adult bone marrow, the expression of *Hmga2* is also increased, and the overexpression of *Lin28b* or *Hmga2* in adult bone marrow ESLAM HSCs significantly increases their self-renewal ability, resulting in higher engraftment in the recipient mice (Copley et al., 2013). This result also indicates that Lin28b overexpression in adult bone marrow HSCs can induce cell autonomous expansion without the fetal liver environment that supports fetal HSC expansion (Agrawal et al., 2024). In addition, *Hmga2* overexpression in human cord blood-derived CD34^+^ HSPCs also enhances long-term engraftment ability by serial transplantation assays (although with erythroid-biased) (Kumar et al., 2019). Conversely, HSCs from *Hgma2* knockout mice exhibit lower self-renewal ability than their counterparts from wildtype mice. These findings indicate that the *Lin28b-let7-Hgma2* axis regulates the self-renewal ability of HSCs in both mice and humans.

However, the mechanism by which *Hmga2* increases the self-renewal ability of HSCs is still not well known. To address this question, Sashida’s group generated Rosa locus *Hmga2* conditional knock-in (KI)-GFP mice to induce *Hmga2* by tamoxifen injection (Sun et al., 2022). They induced *Hmga2* expression in adult hematopoietic cells by tamoxifen injection and transplanted LSK cells from the *Hmga2-GFP*-induced and control (YFP-conditional KI) bone marrow cells into the lethally irradiated congenic mice. Interestingly, 10–12 months after transplantation, whereas the frequency and number of donor-derived HSPC populations and spleen lymphocytes showed similar numbers, *Hmga2-GFP^+^* proportions were significantly increased compared to control YFP-KI mice, indicating that *Hmga2* expression increased HSPC population while still allowing for differentiation to the mature cells. Furthermore, these *Hmga2*-overexpressed HSCs showed elevated *Igf2bp2* expression at a similar level as fetal liver HSCs. The Sashida group also generated mutant *Hmga2*-KI mouse lines that lack the N- and C-terminal domains and found that *Hmga2* requires its three AT-hooks and the C-terminal linker region to activate the transcription of *Igf2bp2* and promote the self-renewal function of HSCs (Sun et al., 2022).

The Sashida group further examined HSC function in mice transplanted with *Hgma2*-KI or control bone marrow cells under stress conditions caused by 5-FU (Kubota et al., 2024). After 5-FU injection, they found increased frequency of *Hmga2-*KI lin-EPCR^+^CD48^−^CD150^+^ HSCs and megakaryocyte progenitors. They also showed that *Hmga2-*KI HSCs went into cell cycle more in the S/G2M phase and divided more compared to WT HSCs in the recipient mice. Three days after 5-FU injection, *Hmga2*-KI HSCs highly expressed *Igf2bp2* and increased cell cycle regulators, but maintained stem cell genes, while repressing genes involved in inflammation and differentiation. Moreover, the Hmga2 protein was phosphorylated by casein kinase 2 (CK2) and promoted to its access and binding to chromatin transcription of anti-inflammatory target genes under stress conditions. These data indicate that *Hmga2* can regulate hematopoiesis under stress conditions. However, *Hmga2* is not expressed in adult HSCs in the physiological setting, so it is not clear if this *Hmga2* function occurs during fetal development, or if *Hmga2* is upregulated and functions under stress conditions even in adult wildtype mice. Indeed, the *Hmga2* protein was upregulated in WT HSCs 3 days after 5-FU injection.

As noted above, *Igf2bp2* is an important co-factor of Lin28 b (Zhu et al., 2011; Shyh-Chang and Daley, 2013). Overexpression of *Hmga2* upregulates *Igf2bp2* expression in mouse bone marrow HSCs (Sun et al., 2022; Kubota et al., 2024). The role of *Igf2bp2* in HSCs has recently been emphasized by several papers (Suo et al., 2022; Yin et al., 2022), especially as it relates to self-renewal ability of HSCs. Since N^6^-methyladenosine (m^6^A) is the most abundant modification on mRNA, Zhang’s group extensively investigated its role in adult hematopoiesis (Yin et al., 2022). They found higher levels of m^6^A in long-term (LT)-HSCs compared to other progenitor populations. While m^6^A and mRNA levels were negatively correlated across hematopoietic progenitor populations, LT-HSCs displayed positive correlations between m^6^A and mRNA levels. It has been reported that *Igf2bp1/2/3* stabilize m^6^A-tagged mRNAs (Huang et al., 2018). As *Igf2bp2* was highly expressed in LT-HSCs, Zhang’s group examined the effects of *Igf2bp2* deletion on HSCs. In *Igf2bp2* ^*−/−*^ mice, the frequency and numbers of LT-HSCs and short-term (ST)-HSCs were significantly decreased. The cell cycle of these *Igf2bp2* knockout HSCs was shifted from G0/G1 quiescent status to cycling. The repopulation ability of *Igf2bp2* ^−/−^ bone marrow was significantly reduced over serial transplantation (Yin et al., 2022). The Zhang group also showed that Igf2bp2 directly binds to its targets, Bmi1, Cdk13, and cbfb in m^6^A sites. Although loss of *Igf2bp2* did not affect any transcripts of *Bmi1,* an essential polycomb repressive complex (PRC) one protein gene for HSC self-renewal, it substantially impaired mRNA stability in HSCs, resulting in reduced *Bmi1* expression, which then derepressed mitochondrial genes and induced loss of HSCs. Thus, *Igf2bp2* seems to play a critical role in stabilizing HSC self-renewal genes.

The role of *Igf2bp2* in aging of HSCs has also been reported (Suo et al., 2022). Suo et al. found that expression of *Lin28b*, *Hmga2*, and *Igf2bp2* in HSCs (CD150^+^CD34^−^LSK cells) was decreased with age, while the expression of *Hmga2* and *Igf2bp2* was maintained in the multi-potent progenitor (MPP) population with age. HSCs from *Igf2bp2* ^*−/−*^ young mice had significantly reduced repopulation ability after transplantation, whereas HSCs from *Igf2bp2* ^*−/−*^ old mice displayed the same repopulation ability as WT HSCs. *Igf2bp2* ^*−/−*^ young HSCs showed lower mitochondrial respiration compared to *Igf2bp2* ^*−/−*^ old HSCs. Suo et al. also demonstrated that *Igf2bp2* deletion significantly reduced the age-related HSC expansion generally seen in WT aged mice (Yamamoto et al., 2018). These data indicate that Igf2bp2 stabilizes HSC self-renewal genes in young mice. One caveat is that this report by Suo et al. used 3-6-month-old mice and defined them as “young mice,” though 6-month-old mice may be considered middle aged. Since the expression level of *Lin28b* in adult mice is significantly less than it is in fetal liver HSPCs by 3 months, it is not clear if *Igf2bp2* is really downstream of *Lin28b* in adult mice.

While *Lin28b* overexpression in adult HSCs induces fetal-type hematopoiesis in the recipient mice, *Hmga2* overexpression in adult HSCs does not (Copley et al., 2013). These data raise the question of what other *Lin28b* co-factors are involved in inducing fetal-like hematopoiesis. Iwama’s group found that the polycomb protein gene *Exh2* represses fetal gene expression in adult bone marrow HSPCs (Oshima et al., 2016). They transplanted E14.5 fetal liver cells from CreERT2 control, *Cre-ERT2: Ezh2f/f*, and *Cre-ERT2: Ezh2f/f: Tet2f/f* embryos into lethally irradiated congenic mice and deleted the *Ezh2* gene by tamoxifen injection 1 month after transplantation. *Ezh2* deletion upregulated *Lin28b*, *Hgma2*, and *Igf2bp3* in LSK HSPC populations in the recipient mice. *Ezh2* deletion also induced more fetal-type hematopoiesis compared to control WT donor cells, including more B-1a, marginal zone B, and TCRgd T cell production in the adult recipient mice. Iwama’s group also conducted an analysis of H3K27me3 levels in WT fetal liver and bone marrow LSK cells and found that fetal HSPC genes are direct targets of Ezh2 in the adult bone marrow, with Ezh2 silencing these genes in the adult bone marrow via H3K27me3 modification (Oshima et al., 2016).

**FIGURE 2:**
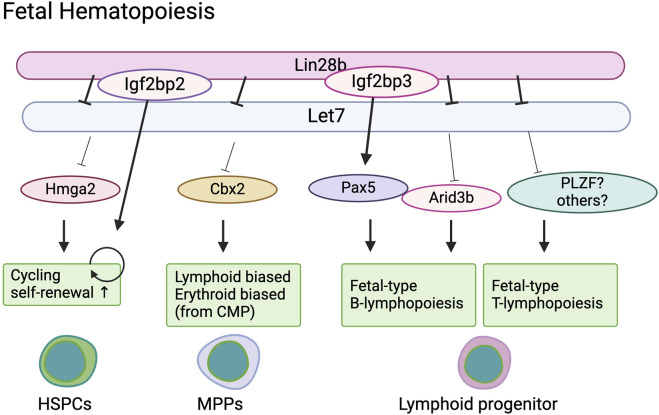
Lin28b regulate fetal hematopoiesis in a stage specific manner.

Rowe’s group found that *Cbx2*, a component of PRC1 and a target of *Let-7*, is also highly expressed in E14.5 fetal liver HSCs and common myeloid progenitors (Wang et al., 2022). *Cbx2* deletion results in diminishment of ST-HSC and MPP2 populations in the fetal liver and smaller numbers of B cells in the neonatal spleen. Conversely, activation of *Cbx2* in adult HSPCs induces erythroid skewing in granulocyte-erythroid-macrophage-monocyte (GEMM) colony-forming cells, similar to the phenotype of fetal GEMM colonies. These results indicate that *Cbx2* plays a role in the differentiation process of fetal HSPCs, possibly under the regulation of *Lin28b* (Figure 2).

## The role of Lin28b in the different hematopoietic lineages

As mentioned above, *Lin28b* expression in adult HSPCs induces fetal-type hematopoiesis upon transplantation. *Lin28b* ^+^ HSCs efficiently repopulate peritoneal B-1a cells and marginal zone B cells, TCRγδ T cells, and iNKT cells, which is similar to the engraftment phenotype seen in yolk sac transplantation or “transient HSC” transplantation (Yoshimoto et al., 2011; Yuan et al., 2012; Beaudin et al., 2016). Although it would be of interest to increase *Lin28b* expression to enhance HSC self-renewal for clinical applications, it is crucial to first obtain a complete understanding of how *Lin28b* expression in adult HSCs alters hematopoiesis. Below, we outline some of the key studies that describe how *Lin28b* changes the output of immune cells generated from adult HSCs.

### B lymphopoiesis by Lin28b-expressing HSPCs

The developmental switch of B cells has been studied for decades (Hardy and Hayakawa, 1991). It was originally described within the context of a layered immune system in which distinct types of stem cells produced different types of lymphoid cells with age (Herzenberg, 1989). Recent data have validated the layered immune system and shown that in the fetal period, rather than different types of stem cells, different types of progenitors exist. Among B cell subsets, B-1 cells and a portion of marginal zone (MZ) B cells are exclusively derived from fetal progenitors (Montecino-Rodriguez et al., 2006; Yoshimoto et al., 2011; Kobayashi et al., 2014; Ghosn et al., 2019). B-1 cells are innate-like B cells that secrete natural IgM antibodies without T cell help and are considered to have the ability to self-renew, maintaining themselves for life without being replenished by bone marrow progenitors. B-1 cells and MZ B cells originate from the yolk sac and embryo as early as E8.25 (Yoshimoto et al., 2011).

When adult HSPCs that are retrovirally overexpressed with *Lin28b* are transplanted into lethally irradiated mice, the *Lin28b*-expressing HSPCs predominantly repopulate B-1a cells and MZ B cells, similar to fetal liver HSPCs (Yuan et al., 2012; Wang et al., 2019). These effects are accompanied by *Let-7* downregulation. Adult HSCs show high levels of *Let-7* and no expression of *Lin28b.* Based on these studies, Zou et al. overexpressed *Let-7* in B progenitors in the fetal liver and converted their fate into adult-type B-2 cells (Zhou et al., 2015). They also overexpressed *Lin28b* in B progenitors in the adult bone marrow and switched their fate into fetal-type B-1a cells. Furthermore, they identified *Arid3a* as a target of *Let-7* that regulates fetal B cell development. Arid3a is part of the AT-rich interaction domain (ARID) super family of DNA-binding proteins and is known to enhance Ig heavy chain expression (Schmidt et al., 2009). *Arid3a* deletion results in embryonic lethality with hematopoietic defects at E12.5 (Webb et al., 2011). Zhou et al. found that the 3′ untranslated region (UTR) of the *Arid3a* mRNA contains several Let-7 target sites and that *Arid3a* knockdown reprograms fetal liver B progenitors to differentiate into adult type B-2 cells. Thus, *Lin28b* regulates B cell fate via the *let-7-Arid3a* axis in early lymphoid/B progenitor stages.

*Lin28b* also plays a role in positive selection of B cells (Vanhee et al., 2019). Vanhee et al. found a correlation between Lin28b expression levels and CD5 expression in B-1 cells. By using doxycycline inducible Lin28b transgenic mice (iLin28b), they found that inducing Lin28 in adult B cell progenitors can generate fully functional B-1 cells via positive selection.

Interestingly, *Lin28b* has also been shown to regulate B cell fate in a *Let-7* independent manner. Wang et al. identified a possible *Lin28b* co-factor in the context of fetal B cell development (Wang et al., 2019). They found that *Igf2bp3,* another RNA binding protein, is highly expressed in fetal liver HSCs, common lymphoid progenitors (CLPs), and pro-B cells, but not in their adult counterparts. Gene expression profiling has identified *Pax5* as a common target of *Lin28* and *Igf2bp3*. *Pax5* mRNA harbors a prominent binding site shared by LIN28 and IGF2BP3 in its 3′UTR near the stop codon and is not predicted to be a *Let-7* target. Wang et al. demonstrated that co-binding of Lin28b and IGF2BP3 to *Pax5* 3′UTR is necessary to fully increase *Pax5* expression (Wang et al., 2019). Pax5 is highly expressed in fetal CLPs compared to adult BM CLPs and has previously been shown to be differentially required for B cell development in the fetal liver and adult bone marrow. *Pax5* deletion results in the absence of B lymphopoiesis in the fetal liver, whereas B progenitors (but not mature B cells) are present in the adult bone marrow (Nutt et al., 1997). Therefore, while *Pax5* is indispensable for fetal B cell lineage commitment, in adult bone marrow, *Pax5* is dispensable for B cell lineage commitment but essential for B cell maturation. These results support the recent notion that fetal B progenitors originate from different progenitors than adult HSCs (Ghosn et al., 2016; Kobayashi et al., 2023). Taken together, *Pax5* is indispensable for fetal-derived B cell development under the control of *Lin28b* and *Igf2bp3* (Figure 2).

One caveat of these studies is whether *Lin28b* overexpression in adult HSC really change the fate of their differentiation at HSC level. The report by Wang et al. showed that *Lin28* and *Igf2bp3* cooperatively stabilize their shared target (*Pax5*) in a *Let-7* independent manner and indue fetal-type B cells, whereas *Lin28b* expand HSCs via *Let-7* downregulation. Therefore, it is plausible that *Lin28b* overexpression induce fetal B-lymphopoiesis in CLP and may not change the differentiation characteristics of HSC itself. For example, if *Lin28b* is transiently expressed only in LT-HSCs, the progenies may not show fetal-type hematopoietic phenotype. Since many of these studies are conducted using HSPCs, not purified LT-HSCs, further investigation would be required to understand how Lin28b works in each early HSPC population such as LT-HSCs, MPPs, CLP, and pro-B cells.

### T lymphopoiesis by Lin28b-expressing HSCs

*Lin28b* can also be used to shape the production and function of T cells. Similar to the ontogeny of B cells, *Lin28b* enables adult progenitors to give rise to certain subsets of T cells that are typically only made by fetal and neonatal progenitors. For example, γδT cells are primarily made in the fetal thymus prior to birth. However, *Lin28b* can reprogram human cord blood CD34^+^ HSPCs to make fetal gamma delta T cells (Tieppo et al., 2020). Similar studies have been performed in mice. However, overexpression of *Lin28b* in adult HSPCs leads to increased numbers of some but not all subsets of murine γδT cells (Yuan et al., 2012). Specifically, adult bone marrow HSPCs transduced with *Lin28b* give rise to the perinatally-derived Vγ1^+^Vδ6.3^+^ T cells but not the fetal-derived Vγ5 dendritic epidermal T cells (DETC) or Vγ6^+^ IL-17 producing γδT cells (Heilig and Tonegawa, 1986; Itohara et al., 1990; Haas et al., 2012; Yuan et al., 2012). Although it is not known why *Lin28b* only permits the production of certain subsets of gamma delta T cells, it is possible that the earliest waves of fetal gamma delta T cells require not only fetal programming by *Lin28b*, but also a fetal thymic environment (Havran and Allison, 1990).

Another subset of T cells that is primarily made in early life is the invariant natural killer T (iNKT) cells, which are an innate subset of T cells that recognize lipids. Previous work has shown that iNKT cells are more abundantly made by fetal and neonatal progenitors in the murine thymus (Lee et al., 2010; Watarai et al., 2010). However, overexpression of *Lin28b* in adult HSPCs facilitates the production of larger numbers of iNKT cells, presumably by enhancing the expression of a key transcription factor, PLZF (promyelocytic leukemia zinc finger), which is required for their development (Yuan et al., 2012; Pobezinsky et al., 2015). Notably, PLZF is also required for the production of Vγ1^+^Vδ6.3^+^ T cells in early life but is targeted by *Let-7* during later stages of development (Kreslavsky et al., 2009; Alonzo et al., 2010; Lu et al., 2015). Thus, overexpression of *Lin28b* may block the repression of PLZF by *Let-7*, allowing for the outgrowth of both Vγ1^+^Vδ6.3^+^ T cells and iNKT cells by adult progenitors (Figure 2).

Alternatively, *Lin28b* may bias T cell output towards the more innate-like lineages (e.g., Vγ1^+^Vδ6.3^+^ T cells and iNKT cells) by altering the T cell receptor (TCR) repertoire. In early life, T cells are made in the absence of terminal deoxynucleotidyl transferase (TdT), which is responsible for the insertion of random nucleotides in the junctional regions of the TCRs (Gilfillan et al., 1993; Komori et al., 1993; Gavin and Bevan, 1995; Cabaniols et al., 2001). As a result, the T cells produced during fetal and neonatal stages of development are less diverse and comprised of shorter and more germline-encoded TCRs. Importantly, recent studies have found that overexpression of Lin28 b in adult progenitors leads to a decrease in TdT expression (Wang et al., 2019; Tieppo et al., 2020). It is possible that innate lymphocytes with germline-configured TCRs are more efficiently made in *Lin28b*-expressing progenitors because the addition of random nucleotides is suppressed (Aono et al., 2000).

In addition to altering the types of T cells made, *Lin28b* also changes the functions of conventional CD8^+^ and CD4^+^ T cells. Interestingly, inducing *Lin28b* in adult progenitors allows them to give rise to CD8^+^ T cells that are phenotypically and functionally analogous to neonatal CD8^+^ T cells (Wang et al., 2016; Wells et al., 2017; Smith et al., 2018; Tabilas et al., 2019). For example, prior to infection, both neonatal and adult *Lin28b* transgenic mice contain a large proportion of CD8^+^ T cells that exhibit a virtual memory (VM) phenotype (CD44^+^CD122^+^), whereas only a small fraction of adult CD8^+^ T cells show this phenotype (Wang et al., 2016). Importantly, intrathymic injections and mixed bone chimeras indicate that the accumulation of VM cells in neonatal mice and Lin28b transgenic mice does not come as a result of changes in the thymic environment, but instead is driven by cell-autonomous changes in the progenitors (Wang et al., 2016). Also, neonatal and *Lin28b* transgenic CD8^+^ T cells preferentially give rise to short-lived effectors after infection, while adult CD8^+^ T cells are more efficient at generating memory precursor cells (Wang et al., 2016; Tabilas et al., 2019). More recently, neonatal and Lin28b Tg CD8^+^ T cells were found to have an enhanced ability to respond to inflammation and protect the host against unrelated infections in a TCR-independent manner, which corresponded to their unique ability to rapidly undergo chromatin remodeling (Watson et al., 2024). Thus, *Lin28b* can be employed to generate CD8^+^ T cells with more fast-acting innate-like properties.

In the case of CD4^+^ T cells, *Lin28b* may bias CD4^+^ T cells towards becoming Tregs in early life (Mold et al., 2010; Wang et al., 2010; Jain, 2020). Notably, inhibition of *Lin28b* in human fetal CD4^+^ T cells impairs their ability to differentiate into regulatory T cells (Bronevetsky et al., 2016). Although the underlying mechanisms are poorly understood, some evidence indicates that *Lin28b* promotes a fetal Treg bias by enhancing TGF-β signaling, which is repressed by let-7 during later stages of development (Bronevetsky et al., 2016). Alternatively, it is possible that *Lin28b* enhances the propensity for naïve T cells to become Tregs by altering thymic selection. Earlier studies have shown that fetal and neonatal T cells in both mice and humans exhibit higher self-reactivity than their adult counterparts, which is a key feature of regulatory T cells (Dong et al., 2017). It will be important to determine whether Lin28b alters the threshold of self-reactivity in T cells, similar to how Lin28b enhances the selection of more self-reactive B cells (Vanhee et al., 2019; Xu et al., 2020).

### Lin28b expressed HSC-derived erythroid-, megakaryocytes-, and myeloid progenitors

*Lin28b* also regulates erythro-myeloid differentiation (Rowe et al., 2016). Lin28b is highly expressed in common myeloid progenitors (CMPs) in the fetal liver. Hematopoietic colonies produced by fetal CMPs are biased towards erythroid outputs, whereas colonies produced by adult BM CMPs are biased towards myeloid outputs with much higher expression of *Let-7*. *Hmg2a* induction in adult CMPs recapitulated erythroid output, suggesting that the *Lin28b-let7-Hmg2a* axis regulates CMP differentiation in the fetal liver.

*Lin28b* and *Hmg2*a are also expressed in megakaryocyte progenitors in the fetus. Fetal-activated platelets express low levels of P-selectin, which is phenocopied when *Lin28b* is overexpressed in adult HSPCs, indicating that the *Lin28b-let7-Hmg2a* axis also controls platelet maturation from fetal megakaryocytes (Stolla et al., 2019).

There is also data to suggest that *Lin28b* induces fetal-type erythropoiesis in humans. Similar to what has been observed in mice, *Let-7* has been associated with a fetal-to-adult switch of globin chains in the human embryo. Specifically, erythrocytes produce a fetal γ-globin-based HbF hemoglobin and transition to an adult β-globin-based HbA hemoglobin prior to birth, and this phenotype is maintained into adulthood (Noh et al., 2009). During the transition, Lin28b is highly expressed in erythrocytes through fetal maturity and exerts the same downregulatory effect on Let-7, resulting in increased expression of HbF (Yuan et al., 2012; Lee et al., 2013). In contrast, *Lin28b* knockdown in cord blood CD34^+^ cells has been shown to decrease HbF and increase HbA. Retroviral overexpression of LIN28b in human adult erythroblasts forces increased production of fetal γ-globin mRNA, with a corresponding decrease in adult β-globin (Lee et al., 2013). These changes seem to be controlled by the inhibition of Let-7 and its effect on BCL11A, a regulator of fetal HbF and adult HbA production (Sankaran et al., 2008; Lee et al., 2013).

Another way in which *Lin28b* exerts its effects on fetal hemoglobin production is through the previously discussed IGF2 family. When overexpressed via retroviral transduction into human CD34^+^ HSPCs derived from adult bone marrow, *IGF2BP1* triggered a robust switch in production of β-globin mRNA to γ-globin mRNA, which coincided with posttranscriptional repression of *BCL11A* (de Vasconcellos et al., 2017). This effect was independent of any increase in Lin28b or inhibition of *Let-7*. The post-transcriptional regulation of *BCL11A* demonstrates a different mechanism of regulation than the transcriptional regulation of *Lin28b-IGF2BP3* binding of the *Pax5* 3′ UTR (Wang et al., 2019), further demonstrating the diverse ways that Lin28b fine-tunes gene expression.

Lin28b also regulate mast cell expansion. Using doxycycline-inducible *Lin28b* transgenic mice, Wang et al. induced ectopic Lin28b expression and found increased numbers of mast cells and their progenitors (Wang et al., 2015). They also found that *Lin28b* expression was upregulated in the pathogenic mast cells from patients with systemic mastocytosis. These results are supported by the recent papers showing that mast cells are fetal-derived (Gentek et al., 2018; Li et al., 2018; Yoshimoto et al., 2022).

## Discussion

We introduced the roles of *Lin28b* and their downstream factors in HSC self-renewal and various lineage specifications. Especially, the *Lin28-let7-Hmga2-Igf2bp2* axis is important for enhancing the ability of HSCs to self-renew. These genes push HSCs to enter cell cycle and proliferate, while still maintaining both stem cell and differentiation abilities via epigenetic modification or m^6^A modifications. However, to utilize the physiological expansion ability of HSCs for clinical applications, thorough consideration is required. For example, all these studies testing the role of *Lin28b* and downstream factors in HSCs use adult bone marrow HSPCs in inducible or knockout mouse models or overexpression of genes by virus transduction. Therefore, it is not clear that *Lin28-let7-Hmga2-Igf2bp2* axis induce HSC expansion in the fetal liver in the physiological setting by the mechanisms described in those reports.

A promising consideration when inducing *Lin28b* expression in adult HSCs is that ectopic transient expression of *Lin28b* will lead to fetal-like hematopoiesis. Since adult HSCs lack the differentiation capability of innate immune cells that are primarily made in early life, induction of *Lin28b* may help to restore or elevate the presence of these immune cells in patients undergoing conventional blood stem cell transplantation therapy or in older people, later in life, who have lost such cells. For example, fetal-derived B-1 cells are known to produce anti-PC antibodies that can prevent pneumococcal invasion; *streptococcus* pneumoniae are among the pathogens that can cause severe infection in older people. Thus, expanding stem cell numbers and regulating immune cell production via *Lin28* could improve the outcomes of both older patients and those receiving transplantation therapy.

A possible negative outcome of *Lin28b* induction into adult HSCs is the potential for developing leukemia, as *Lin28* is highly expressed in some pediatric leukemias (Helsmoortel et al., 2016a; Helsmoortel et al., 2016b). It has also been reported that ectopic *Hmga2* expression in adult HSPCs led to the development of acute myeloid leukemias (AML) in a *Tet2*-deficient context (Bai et al., 2021). Therefore, if one overexpresses Lin28b in HSCs, transient expression would be desired. On the other hand, *Lin28* expression has been reported to promote differentiation of MLL-fusion-driven AML (Eldeeb et al., 2023; Li et al., 2024). Silencing *Hmga2* has also been proposed as a potential therapeutic option for AML (Tan et al., 2018; Yang et al., 2019). As such, the role of *Lin28b* is context dependent, therefore, a better understanding of the mechanisms and roles of the *Lin28b/Hmga2-Igf2bp2/3* axis in different HSPC population at different ages will open the door to more therapeutic opportunities.

In addition, although transplantable HSC numbers are considered to increase during development, a recent report has suggested that HSCs do not robustly expand, as previously thought (Ganuza et al., 2022). It has been postulated that pre-HSCs mature into transplantable HSCs between E11 and E12 (and possibly later) in the fetal liver. However, the mechanisms and processes behind maturation of pre-HSCs into transplantable HSCs are still largely unknown. Therefore, more precise elucidation of HSC numbers and behaviors during fetal life will be required to understand the biological mechanisms of HSC expansion in a physiological setting.

Lastly, there are limitations to studying human fetal HSCs due to different legal allowances for the use of human fetal tissues in each country. Because of this, it may be challenging to understand the mechanisms that support HSC expansion in the human fetal liver or possibly other fetal tissues.

All of these studies are expected to contribute to our basic understanding of hematopoiesis and immune development at different stages of life.
